# Tomatidine enhances lifespan and healthspan in *C. elegans* through mitophagy induction via the SKN-1/Nrf2 pathway

**DOI:** 10.1038/srep46208

**Published:** 2017-04-11

**Authors:** Evandro F. Fang, Tyler B. Waltz, Henok Kassahun, Qiping Lu, Jesse S. Kerr, Marya Morevati, Elayne M. Fivenson, Bradley N. Wollman, Krisztina Marosi, Mark A. Wilson, Wendy B. Iser, D. Mark Eckley, Yongqing Zhang, Elin Lehrmann, Ilya G. Goldberg, Morten Scheibye-Knudsen, Mark P. Mattson, Hilde Nilsen, Vilhelm A. Bohr, Kevin G. Becker

**Affiliations:** 1 Laboratory of Molecular Gerontology, National Institute on Aging, National Institutes of Health, Baltimore, MD 21224, USA; 2 Institute of Clinical Medicine, University of Oslo and Akershus University Hospital, 1478 Lørenskog, Norway; 3 Danish Center for Healthy Aging, University of Copenhagen, Blegdamsvej 3B, 2200 Copenhagen, Denmark; 4 Laboratory of Neurosciences, National Institute on Aging, National Institutes of Health, Baltimore, MD 21224, USA; 5 Laboratory of Genetics and Genomics, National Institute on Aging, National Institutes of Health, Baltimore, MD 21224, USA

## Abstract

Aging is a major international concern that brings formidable socioeconomic and healthcare challenges. Small molecules capable of improving the health of older individuals are being explored. Small molecules that enhance cellular stress resistance are a promising avenue to alleviate declines seen in human aging. Tomatidine, a natural compound abundant in unripe tomatoes, inhibits age-related skeletal muscle atrophy in mice. Here we show that tomatidine extends lifespan and healthspan in *C. elegans*, an animal model of aging which shares many major longevity pathways with mammals. Tomatidine improves many *C. elegans* behaviors related to healthspan and muscle health, including increased pharyngeal pumping, swimming movement, and reduced percentage of severely damaged muscle cells. Microarray, imaging, and behavioral analyses reveal that tomatidine maintains mitochondrial homeostasis by modulating mitochondrial biogenesis and PINK-1/DCT-1-dependent mitophagy. Mechanistically, tomatidine induces mitochondrial hormesis by mildly inducing ROS production, which in turn activates the SKN-1/Nrf2 pathway and possibly other cellular antioxidant response pathways, followed by increased mitophagy. This mechanism occurs in *C. elegans*, primary rat neurons, and human cells. Our data suggest that tomatidine may delay some physiological aspects of aging, and points to new approaches for pharmacological interventions for diseases of aging.

Aging is becoming an international challenge to healthcare systems in both developed and developing countries. Unveiling the most common underlying causes of aging will permit the development of safe interventional strategies to delay aging and extend healthspan in humans^1^,^2^. Several cellular alternations that may be critical towards the development of an aging phenotype been identified, including mitochondrial dysfunction, oxidative stress, genomic instability, and loss of proteostasis^3^. Research regarding mitochondrial dysfunction and age-related diseases, such as neurodegenerative diseases, have highlighted the importance of mitochondrial homeostasis for healthy aging and disease resistance^4^,^5^,^6^. Mitochondrial homeostasis relies on mitochondrial biogenesis, regulated in part by AMPK and PGC-1a, and by selective removal of dysfunctional or damaged mitochondria by mitochondrial autophagy (mitophagy). Impaired mitophagy may occur in physiological aging, premature aging diseases, neurodegenerative diseases, and sarcopenia^6^,^7^,^8^,^9^.

While the average lifespan has increased markedly over the past two centuries, the number of people living with one or more chronic age-related diseases has also increased, particularly in sedentary and over nourished individuals^10^,^11^. Prominent age-related diseases are diabetes, cardiovascular disease, cancers, arthritis, and Alzheimer’s and Parkinson’s diseases. Studies on a broad array of organisms, including *C. elegans*, flies, mice and humans, have shown that dietary energy restriction and exercise can counteract age-related functional decline of cells and organs, and can prevent or delay the onset of various age-related diseases^12^,^13^,^14^,^15^. Emerging evidence suggests that energy restriction, fasting, and exercise may increase cellular and organismal resistance to aging and disease by activating multiple signaling pathways that protect against oxidative, metabolic, and proteotoxic stress^16^,^17^. Examples of these signaling pathways include up-regulation of antioxidant enzymes, sirtuins, DNA repair efficiency, trophic factor production, general autophagy, and mitophagy^9^,^16^,^18^,^19^,^20^. These stress-related activities are examples of hormesis, in which moderate levels of biological stress, such as exercise, elicit a biological response that confers resistance to greater amounts of stress^17^,^21^. Interestingly, recent findings suggest that, in addition to energy restriction and exercise, certain ‘noxious’ chemicals in fruits, vegetables and other plants may counteract aging and disease by inducing adaptive (hormetic) stress responses in cells^22^,^23^.

It has been suggested that small compound inhibition of growth hormone/IGF-1 axis, mTOR-S6K pathway, or activation of AMPK, specific sirtuins, or NF-E2 related factor 2 (Nrf2), may improve healthspan in humans^2^,^24^. Interestingly, many of these pathways are directly or indirectly linked to mitochondria biogenesis (sirtuins, AMPK) and mitophagy (mTOR, SIRT1)^2^,^4^,^19^,^25^,^26^. The discovery of phytochemicals that affect these aging-related pathways may extend our understanding of aging mechanisms and point to novel therapeutic interventions that promote healthy aging and counteract disease processes^23^. Tomatoes are a common, nutritious and inexpensive vegetable containing many bioactive compounds^27^. Tomatidine is the aglycone of α-tomatine, a major glycoalkaloid found in unripe tomato fruits, leaves and stems^27^. Green tomatoes have a high content of tomatine of up to 500 mg/kg, while ripe red tomatoes have less than 5 mg/kg^27^. Tomatidine, a metabolite of tomatine, has multiple beneficial biological effects including anti-inflammatory^28^, anti- tumorigenic, and lipid-lowering activities^29^. Recently, tomatidine was shown to increase mitochondrial DNA content, muscle fitness, as well as lower adiposity in a mouse model of skeletal muscle atrophy, suggesting a role in improving muscle healthspan^30^. However, molecular mechanisms underlying this activity of tomatidine is largely unexplored, and broad health benefits, like lifespan, have not been investigated.

Here, we show that tomatidine treatment in *C. elegans* extends lifespan, improves mitochondrial function, and protects muscle function against age-associated deterioration. We found that tomatidine maintains mitochondrial homeostasis by modulating mitochondrial biogenesis and PINK-1/DCT-1-dependent mitophagy in cells of multiple species, including nematodes, rats, and humans. Tomatidine induces mild oxidative stress, activates the SKN-1/Nrf2 pathway and other cellular antioxidant responses, which induces mitophagy.

## Results

### Tomatidine extends lifespan of wild-type *C. elegans*

**Figure 1:**
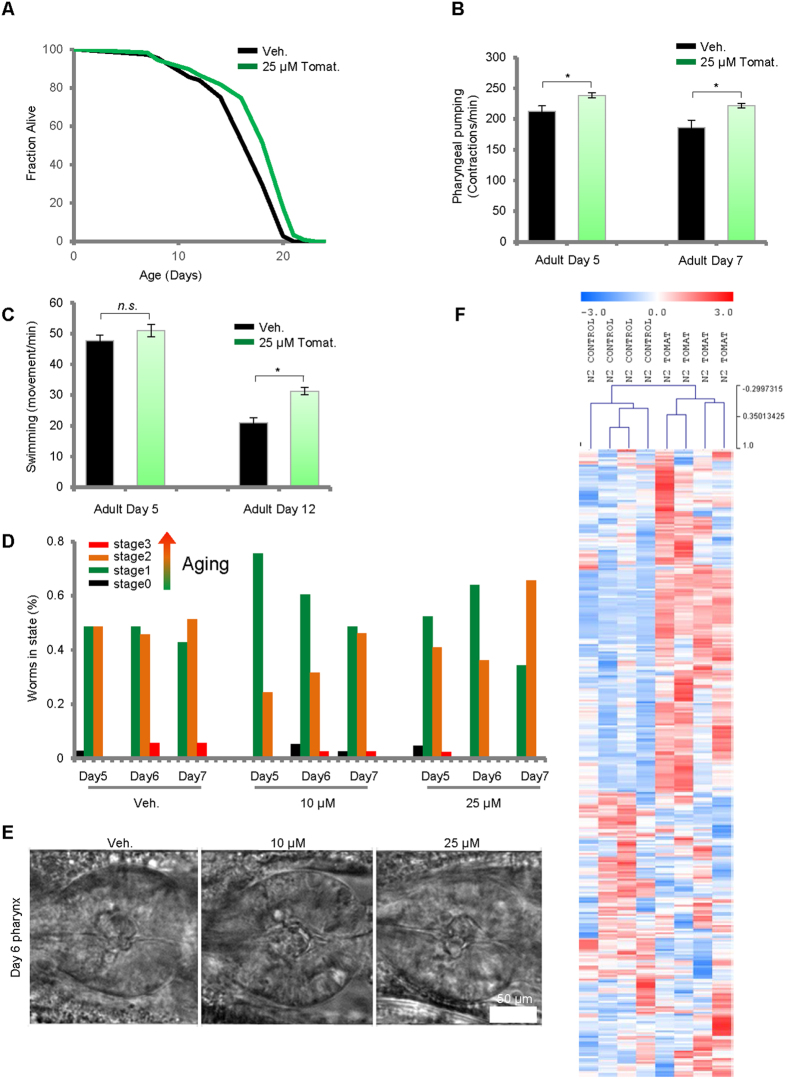
Tomatidine extends lifespan and healthspan in *C. elegans*. (**A**) Lifespans of tomatidine treated *C. elegans* were assessed (n = 91 for vehicle and n = 126 for Tomatine group). (**B**,**C**) Healthspan metrics of pharyngeal pumping (**B**) and swimming movement (**C**) for *C. elegans* treated with 25 μM tomatidine (n = 10–20). (**D**,**E**) Morphology of *C. elegans* pharynx was assessed using machine learning technique to determine age state of pharynx (**D**, n = 30 worms in three separate experiments), with a representative set of pictures shown (**E**). (**F**) Metabolomics showing the relative change in *C. elegans* metabolome with or without tomatidine. Adult D7 worms from tomatidine or vehicle-treated groups were collected and subjected to ALEX-CIS GCTOF mass spectrometry with more than 150 metabolites identified. See Table S1 for a complete list. All bar graphs are expressed as mean ± S.E.M., *p < 0.05; **p < 0.01; ***p < 0.001. See also Figures S1 and S2.

Lifespan of the nematode *C. elegans* quantifies organismal longevity that depends upon various aging processes^31^. We exposed N2 (wild type) *C. elegans* to tomatidine at concentrations of 0, 25, and 50 μM beginning at the L4 stage, and *C. elegans* survival was recorded. Tomatidine consistently extended lifespan at a concentration of 25 μM with (7.0 ± 0.8)% increase (Figs 1A and S1B), while there was no significant change of lifespan at 50 μM (Figure S1A). When assessing the survival curve further, it was noted that many *C. elegans* exposed to 50 μM tomatidine died at relatively early ages (7 to 13 days) compared to *C. elegans* not exposed to tomatidine, but those that survived this period of increased death had a longer maximal lifespan (Figure S1A). This biphasic dose – response effect of tomatidine is consistent with a hormesis-based mechanism of action^17^. We also noticed that 50 μM tomatidine exhibited some toxicity to *C. elegans* healthspan as well (data not shown), while no detectable toxicity was noticed with tomatidine concentrations of 25 μM or less. Based on these dose-dependent responses in lifespan, 25 μM tomatidine was selected as an optimal concentration for most subsequent experiments.

### Tomatidine enhances muscle function and modulates metabolism in *C. elegans*

There is a dramatic physical decline in swimming ability, pharyngeal pumping, and maximum movement velocity as *C. elegans* age^32^,^33^. These behaviors reflect the overall muscle health of *C. elegans,* and any attenuation of these age-related declines due to an intervention would suggest anti-aging effect on muscle cells. Our data consistently showed age-dependent decreases of pharyngeal pumping and swimming movement in N2 *C. elegans* (Fig. 1B,C). *C. elegans* treated with 25 μM tomatidine exhibited enhanced pharyngeal pumping at multiple time points during their life (Fig. 1B). Although 25 μM tomatidine did not affect swimming ability in young (adult day 4) *C. elegans*, as they aged (adult day 12), tomatidine significantly delayed the age-related decrease of swimming movement scores (48% improvement compared with vehicle) (Fig. 1C). Maximum movement velocity in *C. elegans* has been suggested to decline with age and to correlate with longevity^33^. We verified the decrease of maximum movement velocity with age in N2 *C. elegans*, but found no significant differences in maximum movement velocity in tomatidine-treated *C. elegans* from adult days 4 to 10 compared with vehicle treatment (Figure S1C, and data not shown). Collectively, tomatidine is able to alleviate some features of aging-related physical decline in *C. elegans*, which indicates an improvement in healthspan.

We further asked whether the improvement of swimming and pharyngeal pumping implied inhibition of sarcopenia, so we performed phalloidin staining on worm muscle cells to assess actin filament structure^34^. We carefully analyzed the muscle morphology in day 4 and day 8 adult worms treated with or without tomatidine, and presented the data as percentage of muscle cells that possessed “no damage”, “minor damage”, or “major damage” (Figure S1E). Tomatidine had no significant effect on actin filament damage in adult day 4 worms. However, in day 8 worms, tomatidine decreased the percentage of muscle cells that exhibited severe damage: first repeat, 54% in N2 (veh) compared with 24% N2 (tomatidine); second repeat 15% in N2 (veh) vs. 7% N2 (tomatidine) (Figure S1F). Collectively, these data suggest that tomatidine inhibits sarcopenia not only in mice^30^ but also in *C. elegans*.

We further investigated whether tomatidine-induced improvement of muscle-dependent behaviors could be related to the preservation of muscle cells. We previously showed that pharynx muscle morphology reflects the aging state of the whole organism, and based on this mechanism in combination with machine-learning software, we analyzed the effects of tomatidine on age stage scoring of pharynx in accordance to the previous publications^35^,^36^. We defined *C. elegans* pharynx scoring from stage 0 (young) to stage 3 (old): stage 0 *C. elegans* are predominantly found in the first 2 days of adulthood and are developing fecundity. Both Stage-1 and Stage-2 *C. elegans* are actively laying eggs. Stage-3 *C. elegans* are predominantly found after egg laying has ceased, beginning as early as day 7 of adulthood. Administration of 10 μM or 25 μM tomatidine consistently lowered the age-states of individual *C. elegans* pharynx (Figs 1D,E and S1D). Notably, for 25 μM tomatidine-treated worms, there are more stage 2 pharynx compared to the Day 7 vehicle condition, but there are no stage 3 pharynx in this group while stage 3 pharynx is 5% in Day 7 vehicle condition. All these data suggest that tomatidine may attenuate age-related pharynx deterioration. These results suggest that tomatidine likely enhances pharyngeal pumping by preservation of youthful pharynx morphology.

Efficient metabolism is important for health, and decrements in cell metabolism have been implicated in age-related diseases and longevity^37^,^38^. To test the effect of tomatidine on *C. elegans* metabolomes, adult D7 tomatidine or vehicle-treated whole body tissues of *C. elegans* were collected and subjected to ALEX-CIS GCTOF mass spectrometry with more than 150 metabolites identified (Fig. 1F and Table S1). Tomatidine affected the levels of several metabolites, and we chose to focus on metabolites relating to the TCA cycle and amino acid metabolism, due to their importance in energy production and maintenance of muscle mass, and therefore muscle performance. A reduction of many TCA intermediates, including citrate, isocitrate, and malate, coupled with decreased lactate production and steady glycolysis intermediates suggests that tomatidine enhances general levels of oxidative phosphorylation in *C. elegans*. Tomatidine-treated *C. elegans* exhibited increased levels of many free amino acids (leucine, threonine, isoleucine, tryptophan, proline, arginine, histidine, valine, methionine) suggesting an increased catabolism (Figure S2A). Collectively, these results demonstrate that tomatidine affects several metabolic pathways which may be integral to its anti-aging properties.

### Gene expression analysis reveals regulation of mitochondrial pathway by tomatidine

**Figure 2:**
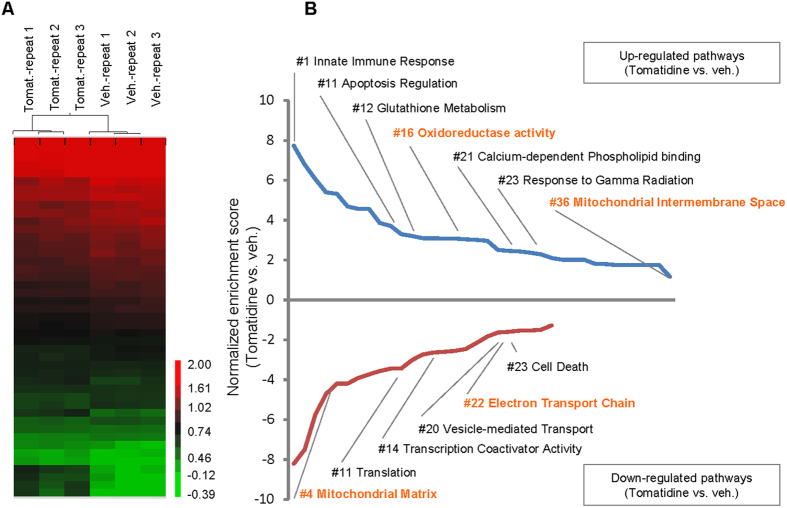
Effects of tomatidine on the transcriptome of *C. elegans*. (**A**) Heatmap of microarray data showing genes that were differentially expressed in control and tomatidine-treated nematodes. (**B**) A list of significantly changed GO signaling pathways in tomatidine-treated worms compared with vehicle-treated changes. Mitochondria-related pathways are marked orange. See Table S2 for a complete gene list.

We next investigated the mechanism(s) by which tomatidine enhances lifespan and muscle-related healthspan. To this end, we performed whole-genome gene expression analysis using microarrays to elucidate potential pathways that might mediate the anti-aging action of tomatidine. Heatmap analysis suggested that tomatidine had significant effects on the profiles of expressed transcripts (Fig. 2A). Gene ontology (GO) analysis identified 36 GO signalling pathways upregulated and 25 GO signalling pathways downregulated (Fig. 2B and Table S2). Intriguingly, tomatidine had significant effects on mitochondria function, as evidenced by changes in several mitochondria-related signaling pathways, including those affecting the electron transport chain, the mitochondrial inner membrane space and matrix, and the metabolism of glutathione (Fig. 2B). Additionally, the GO analysis also suggests the improvement of other health-related signaling pathways, including pathways associated with cell fate regulation, metabolism, ROS regulation, and increased resistance to exogenous stress (response to gamma radiation). These data suggest that tomatidine affects mitochondrial function and ROS metabolism.

### Evidence that tomatidine stimulates mitochondrial biogenesis and mitophagy

**Figure 3:**
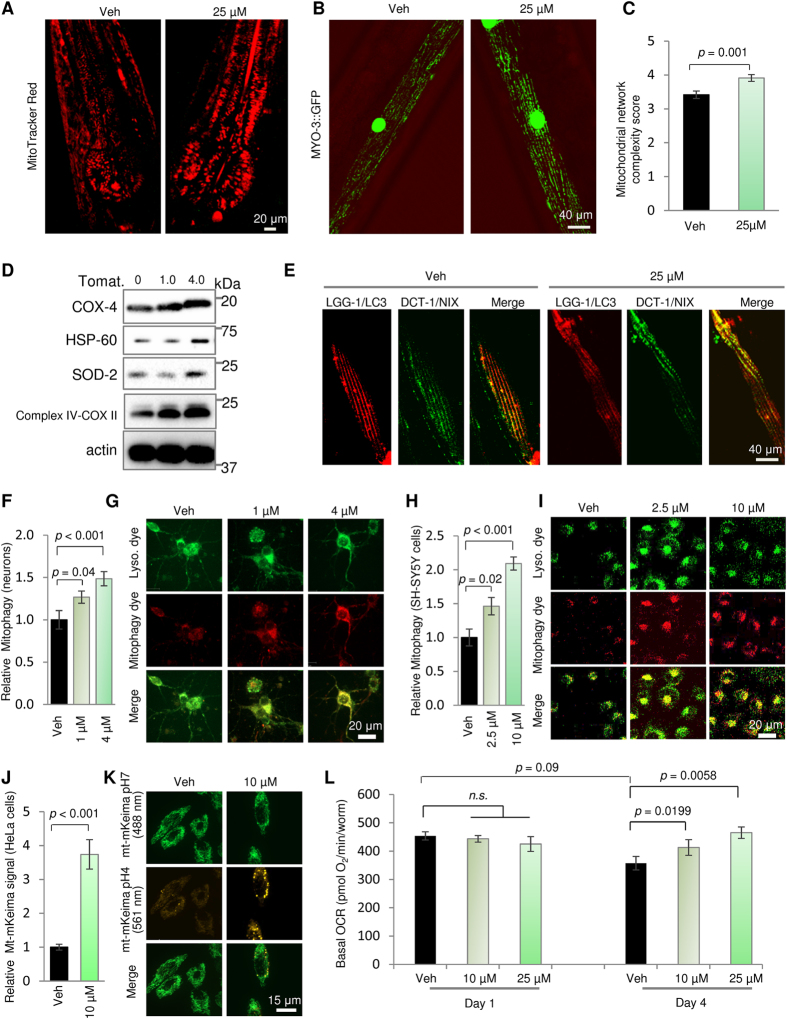
Tomatidine modulates mitochondrial homeostasis by stimulating both mitochondrial biogenesis and mitophagy. (**A**) Assessment of mitochondrial content in nematode pharynx determined by quantifying mitoTracker Red fluorescence (n = 15). Representative images are also shown. (**B**,**C**) Monitoring of mitochondrial network of the pharynx muscle cells in vehicle- and tomatidine-treated *myo-3::*GFP nematodes. Representative images are shown in panel B, and quantification of mitochondrial network scoring is shown in panel C (n = 40). (**D**) Immunoblot showing relative levels of the indicated mitochondrial proteins in control and tomatidine-treated human neural cells. For full-length gels see Figure S4A. (**E**) Detection of muscle cell mitophagy in vehicle- and tomatidine-treated nematodes. The mitophagy-screening strain N2;*Ex(pmyo-3::dsred::lgg-1;pdct-1::dct-1::gfp)* was used for mitophagy detection. A set of representative images is shown. (**F**,**G**) Assessment of mitophagy in vehicle- and tomatidine-treated primary rat cortical neurons. Colocalization between the mitophagy dye and the lysosome dye were quantified (**F**) with a representative set images shown in panel G. (**H**,**I**) Assessment of mitophagy in vehicle- and tomatidine-treated human neural cells. Colocalization between the mitophagy dye and the lysosome dye were quantified (**H**) and representative images are shown in panel I. (**J**,**K**) Evaluation of mitophagy in vehicle- and tomatidine-treated HeLa cells expressing mt-mKeima (see Supplementary Methods for details). Ratios indicating relative levels of mitophagy were quantified (**J**) and representative images are shown in panel K. (**L**) Evaluation of *C. elegans* basal OCR was performed using a Seahorse XF96 instrument (mean ± S.E.M with n = 10 replicates/group). See also Figure S2.

Given the significant changes of tomatidine on mitochondria-related signaling pathways, we examined how tomatidine modulates mitochondrial homeostasis in *C. elegans*, primary rat cortical neurons, and human cells. Since mitochondrial homeostasis is tightly regulated by mitochondrial biogenesis and quality control through mitophagy^5^,^39^, we first determined whether tomatidine changes mitochondrial content and morphology in *C. elegans*. The pharynx is an important organ composed of pharyngeal muscles that show age-dependent loss of function (pumping, Fig. 1B) and structure (Fig. 1E), which are attenuated by tomatidine. We therefore examined mitochondrial distribution in the pharynx using MitoTracker Red. There were more mitochondria in pharyngeal cells in tomatidine-treated *C. elegans* compared with vehicle control (Fig. 3A). We further verified tomatidine-induced mitochondrial biogenesis using a muscle specific *pmyo-3::gfp* strain (*ccIs4251*) (Fig. 3B). Since there is an age-dependent disorganization of mitochondrial network^5^, we took advantage of this *myo-3* strain and used a semi-quantitative rating scale to evaluate the mitochondrial networks in a double-blind manner; the lowest value of one indicates highly impaired mitochondrial network, while a value of five signifies optimally organized mitochondrial network running in parallel with the myofilament lattice (see Methods). Tomatidine moderately increased the mitochondrial network morphology score (control, 3.4; tomatidine, 3.9) (Fig. 3B,C). Tomatidine-induced mitochondrial biogenesis was conserved from *C. elegans* to mammalian cells since treatment of primary rat cortical neurons with 4 μM tomatidine increased their mitochondrial content, mitochondrial membrane potential, and cellular ROS (Figure S2B–D). Tomatidine increased, in a concentration-dependent manner, the expression of several major mitochondrial proteins in a human neural cell line including the inner mitochondrial membrane protein COX-VI, HSP-60 (a mitochondrial protein chaperone), superoxide dismutase 2 (SOD2), and mitochondrial Complex IV-COX II (Fig. 3D). These data show that tomatidine affects mitochondrial content and quality.

In view of the intricate interplay between mitochondrial biogenesis and mitophagy^5^,^39^, we asked if tomatidine affects mitophagy. A series of novel techniques were applied to critically investigate mitophagy. A mitophagy reporter strain in *C. elegans* has been established recently whereby colocalization of LGG-1 (the *C. elegans* homolog of a key mammalian autophagy marker LC3) and mitochondrial outer membrane localized mitophagy receptor DCT-1 (a putative orthologue to the mammalian NIX/BNIP3L) would indicate mitochondria undergoing mitophagy^5^. *C. elegans* were treated with 25 μM tomatidine from the L4 stage, followed by imaging at adult day 4. The data showed that 25 μM tomatidine induced an increase of mitophagy in *C. elegans* muscle cells (Fig. 3E and quantification in S2E). We further asked whether tomatidine can also induce mitophagy in primary rat cerebral cortical neurons and human neural cells. A commercial kit was applied for mitophagy detection in which a pH-dependent increase of fluorescence detects incidence of “mitolysosomes” (which occur when mitochondria in autophagosomes fuse with acidic lysosomes) which can be detected as the colocalization of the ‘Mtphagy’ dye and a lysosome dye (see Methods for details). In line with an induction of mitophagy in *C. elegans*, tomatidine increased mitophagy incidence in rat cortical neurons as indicted by increased colocalization of ‘Mtphagy’ dye with the lysosome dye (Fig. 3F,G). Similarly, tomatidine also induced mitophagy in human neural cells (Fig. 3H,I). Additionally, we used a standard mt-mKeima mitophagy reporter to verify changes of mitophagy by tomatidine; the mt-mKeima exhibits green fluorescence (excitation 488 nm) in healthy mitochondria (pH ~8) and a red signal (excitation 561 nm) when mitochondria are in mitolysosomes^40^,^41^. As expected, tomatidine increased the mt-mKeima signal (561 nm) indicating higher mitophagy (Fig. 3J–K). In conclusion, tomatidine can induce mitophagy across species.

We further explored how tomatidine-induced increases in healthy mitochondria might affect overall mitochondrial respiratory chain function in the entire *C. elegans* by examining basal oxygen consumption rate (OCR) with Seahorse XF extracellular flux instrument. *C. elegans* were exposed to increasing doses of tomatidine from the L4 stage, and basal OCR was measured at adult days 1 and 4. There was a trend towards an age-dependent decrease of OCR between adult day 1 and 4*C. elegans*, and tomatidine increased basal OCR in adult day 4 worms compared age-matched vehicle-treated control *C. elegans* (Fig. 3L), suggesting increased mitochondrial quality. Taken together, these results suggest that tomatidine improves mitochondrial homeostasis through induction of mitochondrial biogenesis and mitophagy, and that this process is conserved across species from *C. elegans* to human cells.

### Tomatidine-induced healthspan improvement requires PINK-1/DCT-1-related mitophagy

**Figure 4:**
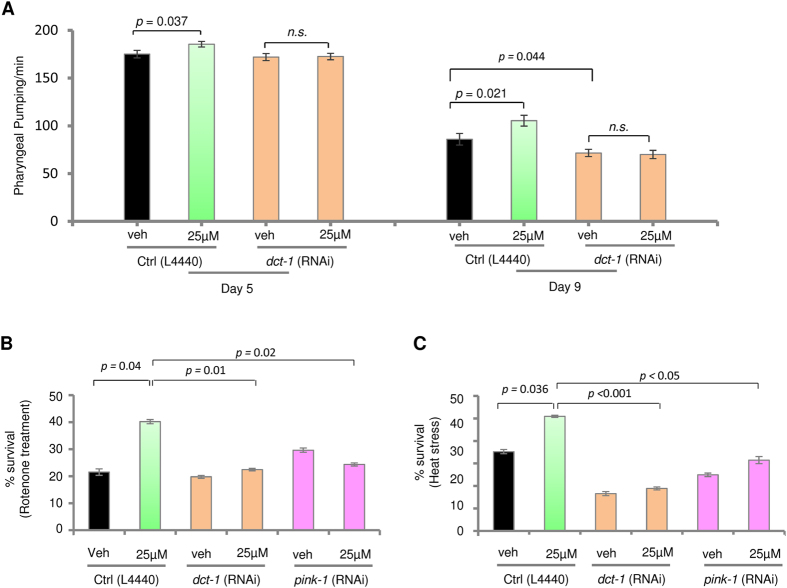
Mitophagy is necessary for tomatidine-induced stress resistance in worms. (**A**) *dct-1* knockout nematodes were treated with 25 μM of tomatidine and pharyngeal pumping was assessed at day 5 and day 9 (mean ± S.E.M., n = 39–40). (**B**) Day 4 nematodes treated with or without tomatidine were exposed to the mitochondrial toxin rotenone (25 μM) for 2 days and survival was quantified on day 6 (mean ± S.E.M, n = 41–61). (**C**) Day 4 nematodes treated with or without tomatidine were exposed to heat stress (37 °C for 7 h), and survival was quantified (mean ± S.E.M, n = 41–61).

Mitophagy plays a primary role in mitochondrial quality control, and impaired mitophagy causes neurodegeneration, dysfunctional cell metabolism, and even premature aging and shorter lifespan in laboratory animal models^4^,^5^,^8^,^9^,^42^. In *C. elegans*, the proteins PINK-1 and DCT-1 are two critical conserved executors of mitophagy, and may act on a similar genetic pathway to induce mitophagy^5^. To investigate the roles of mitophagy in tomatidine-related health benefits in wild type *C. elegans*, we knocked down *dct-1* using RNA interference (RNAi) and examined changes in pharyngeal pumping (Fig. 4A). In older *C. elegans* (day 9), *dct-1* knockdown exacerbated the age-dependent decrease of pharyngeal pumping. Importantly, *dct-1* knockdown abolished tomatidine-induced increase of pumping measured in 5 and 9 day-old adults, unveiling an essential role of mitophagy induction in tomatidine-induced increase of pumping (Fig. 4A).

To further investigate the roles of mitophagy in tomatidine-induced health benefits, we assessed mitophagy-depleted worms for stress resistance. N2 *C. elegans* were sensitive to mitochondrial complex I inhibitor rotenone-induced death with 21% survival. Tomatidine treatment doubled the survival of rotenone-treated worms to over 40% (Fig. 4B). The protective effect of tomatidine against rotenone-induced mitochondrial stress was abolished in both *dct-1-* and *pink-1*-deficient *C. elegans*. Tomatidine treatment also protected wild type *C. elegans*, but not *dct-1-* and *pink-1*-deficient ones, against heat stress (Fig. 4C). These findings indicate that PINK-1/DCT-1-related mitophagy contributes to the health-promoting and stress resistance effects of tomatidine.

### Tomatidine induces mitophagy by activating the SKN-1/Nrf2 pathway

**Figure 5:**
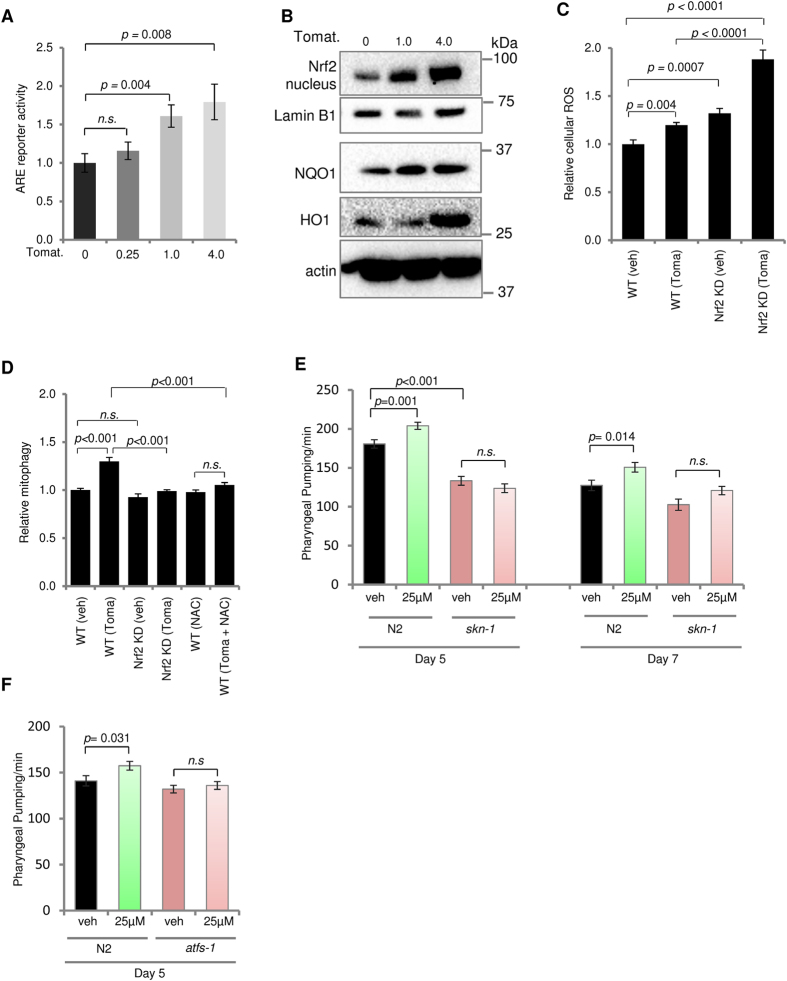
Tomatidine-induced mitophagy is mediated by ROS and activation of Nrf2/SKN-1. (**A**) Assessment of tomatidine-induced Nrf2/ARE activity in ARE-*bla* HEK-293 cells (mean ± S.E.M., n = 6). (**B**) Immunoblot showing effects of tomatidine on levels of the indicated proteins in human neural cells. Cells were treated with the indicated concentrations of tomatidine for 24 h. For Nrf2, the nuclear fraction was isolated and used for immunoblotting. For full-length gels see Figure S4B. (**C**) Changes of cellular ROS in WT and Nrf2 knockdown human neural cells treated with or without 4 μM tomatidine. Cells were stained with DHE (an indicator of superoxide levels) followed by FACS analysis (mean ± S.E.M, n = 6). (**D**) Assessment of mitophagy in WT and Nrf2 knockdown human neural cells treated with or without 4 μM tomatidine (24 h) or a ROS scavenger NAC (1 mM for 3 h) (mean ± S.E.M, n = 6). (**E**) Effects of tomatidine on pharyngeal pumping rate in N2 wild type and *skn-1 (zu135) C. elegans* (mean ± S.E.M, n = 30). See also Figure S3. (**F**) Pharyngeal pumping rate in both N2 wild type and *atfs-1* RNAi knockout *C. elegans* (mean ± S.E.M, n = 20).

To elucidate the signaling pathway(s) by which tomatidine stimulates mitophagy, we used fluorescent reporter techniques and human HEK-293 cells to determine whether tomatidine affected the promoter activity of several transcriptional activators that regulate mitochondrial homeostasis. These include the longevity and autophagy-regulating protein SIRT1, the master regulator of mitochondrial biogenesis PGC-1α, and the anti-oxidant nuclear factor E2-related factor 2 (Nrf2)/antioxidant response element (ARE) pathway 8,26,43. While tomatidine did not affect SIRT1 or PGC-1α promotor activities (Figure S3A,B), tomatidine induced a significant increase of Nrf2/ARE reporter activity in a concentration-dependent manner (Fig. 5A).

Because tomatidine increased Nrf2/ARE reporter activity, we performed immunoblot analysis to measure levels of several proteins encoded by Nrf2-responsive genes in human neural cells exposed to tomatidine. Upon activation by ROS, Nrf2 translocates from the cytoplasm to the nucleus where it binds to the ARE region to transcriptionally activate genes which encode antioxidant proteins, including NAD(P)H quinone oxidoreductase 1 (NQO1) and heme oxygenase 1 (HO1)^43^,^44^,^45^. Human neural cells exposed to tomatidine exhibited increased amounts of nuclear Nrf2, and increased levels of NQO-1 and HO-1 (Fig. 5B), indicating that tomatidine activates the Nrf2-ARE pathway. In control cells with normal levels of Nrf2, tomatidine induced a 20% increase in levels of ROS (Fig. 5C). Basal levels of ROS were increased in Nrf2-deficient cells, and tomatidine caused a large increase in ROS levels in the Nrf2-deficient cells (Fig. 5C). Moreover, tomatidine-induced mitophagy was attenuated in human neural cells in which Nrf2 was knocked down, and in cells treated with the antioxidant N-acetylcysteine (Fig. 5D), indicating that tomatidine stimulates mitophagy by a mechanism involving ROS-dependent activation of Nrf2. Collectively, these data suggest that tomatidine can induce activation of the Nrf2/ARE pathway, contributing to mitophagy induction.

To investigate whether activation of the Nrf2/ARE pathway in mammalian cells is also conserved at the organismal level, we examined the effect of tomatidine on the null *C. elegans skn-1 (zu135*) mutant strain (the *C. elegans skn-1* gene is orthologous to the mammalian *Nrf2* gene). When pharyngeal pumping was assessed, 25 μM tomatidine consistently enhanced pumping rate in adult wild type *C. elegans* (Fig. 5E). However, *skn-1* mutation compromised tomatidine-induced increase of pumping rate, indicating that tomatidine requires Skn-1 activity to enhance muscular function. It is possible that tomatidine may activate other stress response pathways key to mitochondrial stress, such as the mitochondrial unfolded protein response (UPR^mt^). Knocking down *atfs-1*, a key gene in regulating UPR^mt^ expression^46^, compromised tomatidine-induced increase of pumping rate (Fig. 5F). This suggests that UPR^mt^ may play a positive role in tomatidine-induced health benefits. Taken together, these data indicate that tomatidine induces mitophagy and preserves cell function during aging, possibly through multiple stress response pathways, such as the Nrf2/Skn-1 pathway and the activation of UPR^mt^.

## Discussion

Compromised mitochondrial quality and function contributes to biological and pathological aging, and many major aging-related diseases. Accumulation of damaged mitochondria within cells can trigger apoptosis, inflammation, and cell senescence^4^,^6^,^47^. Sarcopenia is a common aging phenotype for which there is no effective therapy, affecting 13–24% of people over 60 years old and increasing to more than 50% in people over 80 years old^30^,^48^. With a growing older population, there is great interest in developing pharmacological approaches to inhibit the progress of sarcopenia and other aging pathologies, thereby promoting healthier aging. Recent studies suggest that pharmacologically improving mitochondrial quality may alleviate muscle atrophy in aged mice^49^,^50^,^51^. We found that tomatidine preserves muscle function during aging, and extends lifespan, in *C. elegans*. Further studies are necessary to dissect the specificity of the beneficial effects of tomatidine in *C. elegans*. For example, it is conceivable that the bacteria upon which the worms feed could metabolize tomatidine to a different compound that, in turn, exerts a beneficial effect on the worms. However, it is important to note that tomatidine improves mitophagy in both *C. elegans* and cultured human cells that lack bacteria. Additionally, it would be interesting to investigate whether other steroids with a similar structure to tomatidine produce similar effects in *C. elegans*.

Our findings suggest that tomatidine may protect *C. elegans* muscle function from age-related deterioration by activating the Nrf2/SKN-1-DCT-1 pathway and up-regulating mitophagy and antioxidant cellular defenses. A recent study in mice provided evidence that tomatidine stimulates the growth of mouse skeletal muscle cells, in part by activating the mTOR pathway^30^. In humans, sarcopenia is a common feature of aging that involves the accumulation of dysfunctional mitochondria and associated deterioration of muscle fiber cells themselves, in combination with infiltration of adipocytes and inflammatory immune cells^52^. In addition, an impaired ability of muscle stem cells to generate new myocytes may be impaired with aging and more so in sarcopenia^53^. All cells that comprise *C. elegans* pharyngeal muscles are postmitotic muscle cells. The beneficial effects of tomatidine in counteracting age-related deterioration of muscle function in *C. elegans* are therefore not the result of effects on muscle stem cells or immune cells. Instead, our findings suggest that tomatidine may influence either the muscle cells themselves, or the nervous system associated with muscle function, and therefore may be particularly relevant to processes occurring within skeletal muscle fiber cells in sarcopenia.

Emerging evidence suggests a pivotal role of mitophagy in health and aging^4^,^5^,^51^, pointing to the possibility that mitophagy induction can improve healthspan and lifespan. We show here, for the first time, that a natural product can not only induce mitochondrial biogenesis, but also enhance the quality of the cellular mitochondrial pool by stimulating mitophagy. The mitophagy induction by tomatidine was stringently evaluated by several mitophagy detection methods targeting the mitophagosome or mitolysosome (Fig. 3). Most importantly, mitophagy is required for tomatidine-induced extension of healthspan and stress resistance. Since we did not detect changes of reporter activities of SIRT1 and PGC-1α in cells exposed to tomatidine, our data suggest that the mitochondrial benefit of tomatidine is dependent on the Nrf2/SKN-1 pathway. However, we do not rule out the possibility that tomatidine may, directly or indirectly, change SIRT1 or PGC-1α activities at post-translational levels, for example by changes in deacetylation activity. Also, this does not rule out other mechanistic pathways that could play a role in tomatidine-dependent improvement of mitophagy. For example, tomatidine may upregulate mitophagy through induction of p62 via the Nrf2/SKN-1 pathway. p62 (also named sequestosome 1/SQSTM1) is a major autophagy receptor that involves in the maturation of autophagosome and the autophagic degradation of polyubiquitin-containing bodies^54^. Recently, it has been shown that a synthetic p62-mediated mitophagy inducer (PMI) can activate mitophagy through upregulation of p62 via the ARE-Nrf-2 pathways^55^,^56^. Thus, future work on the effect of tomatidine on p62 is of interest. In all, our findings suggest that tomatidine stimulates mitophagy by activation of the PINK1/DCT-1 pathway via Nrf2/SKN-1 activation, which is an important pathway for enhancing mitochondrial and muscle health in young and old worms.

Although cellular stress response signaling and mitophagy have both been proposed to be essential in health maintenance, an interplay between Nrf2 and mitophagy in aging has not been established. A recent study found repression of the Nrf2 pathway in a premature aging disease, Hutchinson-Gilford progeria syndrome (HGPS). HGPS is caused by mutation of the nuclear architectural proteins lamin A and C, with HGPS patients showing profound growth delays and premature aging phenotypes, including cardiac muscle and skeletal muscle pathologies^57^,^58^. Compromised Nrf2 activity contributes to premature aging in experimental models of HGPS through increasing chronic oxidative stress, and reestablishment of the Nrf2 pathway using small compound Nrf2 activators ameliorates disease phenotypes^58^. We showed that tomatidine triggers mitophagy and induces Nrf2 activation, and deficiency of either mitophagy or Nrf2/SKN-1 compromises stress resistance and impairs mitochondrial function. Furthermore, we showed a causative relationship between Nrf2 activation and mitophagy induction. In addition, accumulating evidence suggests a signaling role for ROS in the stimulation of mitophagy in cells under mild stress. Studies from yeast and mammalian cells suggest moderately elevated ROS levels can induce mitophagy, which then clears aged or dysfunctional mitochondria. However, if mitophagy is compromised and/or ROS levels are excessively high, mitochondrial dysfunction becomes exacerbated in a vicious cycle^59^,^60^. Thus, growing evidence from both laboratory and clinical studies shows that ROS has a dynamic role in health and disease. While excessive ROS can cause cellular and systemic damage and promote disease, moderately elevated ROS levels enhance cellular stress resistance and protect against disease^17^,^21^. In line with these findings, we show that tomatidine can induce a moderate increase in ROS levels which is necessary to trigger mitophagy.

Many of the chemicals produced in vegetables, fruits and other plants (phytochemicals) that have been found to have beneficial effects on cells and organisms are believed to function as noxious agents (i.e., ‘antifeedants’ or toxins) that dissuade insects and other organisms from consuming the plants^22^. Herbivorous organisms evolved multiple mechanisms to prevent consumption of toxic amounts of the phytochemicals including taste receptors, emesis, enzymes (cytochrome p450s) that rapidly metabolize the phytochemicals, and adaptive stress response pathways in cells throughout the body and brain. Phytochemicals that activate adaptive cellular stress response pathways typically have a bitter taste^22^. Examples of phytochemicals that activate the Nrf2 antioxidant response pathway include sulforaphane, curcumin, and plumbagin^23^. Such noxious phytochemicals are typically concentrated in the parts of plants that are exposed to the environment and/or are involved in seed production including the ‘skin’ of fruits, and developing buds/sprouts. In the case of the tomato fruit, tomatidine is present in high amounts in the unripe green tomato and in much lower amounts in the ripe red tomato. This is consistent with a role for tomatidine in protecting the unripe tomato against consumption, with the reduction in tomatidine levels in the ripe fruit then enabling consumption of the fruit and dispersal of the seeds by the consumer^61^. Our findings suggest that moderate amounts of tomatidine can activate adaptive cellular stress responses in muscle cells or neurons that protect cells against metabolic and oxidative stress, which can counteract age-related dysfunction and degeneration. One prominent response of cells to tomatidine is induction of mitophagy, which preserves cellular function during aging. Because mitochondrial dysfunction and defective mitophagy are implicated in the etiology of several major age-related diseases, including sarcopenia, diabetes, Alzheimer’s disease and Parkinson’s disease, it will be of considerable interest to determine whether tomatidine has beneficial effects in animal models of these disorders.

## Materials and Methods

### *C. elegans* strains and cultivation

*C. elegans* were grown on standard nematode growth medium (NGM) and maintained following standard protocols as described previously^62^. The Bristol strain N2 (wild type), CB5600 *ccIs4251[pSAK2 (myo-3p::gfp-lacz(NLS))* + *psak4 (myo-3p::mitochondrial gfp)*I, and *skn-1(zu135)* were obtained from the Caenorhabditis Genetics Center (CGC). The mitophagy reporter strain *N2;Ex(pmyo-3::dsred::lgg-1; p*_*myo-2*_*GFP)Ex(pdct-1::dct-1::gfp; pRF4)* was from Prof. N. Tavernarakis (University of Crete, Greece)^5^. All *C elegans* were cultured at 25 °C on solid NGM agar plates with OP50 food source unless otherwise noted^62^.

### C. elegans longevity assays

Lifespan analysis was performed as mentioned previously^8^,^37^,^38^. About 8–10 adult D1 *C. elegans* were transferred to 4 plates and allowed to lay eggs for 5 hours to give rise to synchronous population. After hatching, L4 stage nematodes were transferred to respective condition plate with either 10, 25, or 50 μM of tomatidine (all with 50 μM FuDR). Animals were scored as dead or alive every other day. Missing worms were censored from the lifespan count. Significance was determined by log rank test, and mean was calculated by averaging the days of *C. elegans* death for each condition. Figures display a smoothed Kaplan Meier survival curve of the pooled populations.

### Luciferase assays of promoter activities for Nrf2/ARE, SIRT1 and PGC-1α

Luciferase assays were performed as described elsewhere^26^,^43^. The ARE-*bla* construct was used for Nrf2/ARE reporter assay^43^; the PGC-1α-2kb (the Renilla luciferase-expressing plasmid/pRL-TK used as an internal control.) was for PGC-1α promoter activity detection^26^.

SIRT1 promoter activity was detected by a pTA-Luc SIRT1 promoter (-202), a gift from Dr. Toren Finkel^63^. In Brief, plasmids were transfected to HEK-293 cells for 24 h, cells were then treated with different concentrations of tomatidine for 24 h. Luciferase activity was measured using a Dual-Luciferease-Reporter System (Promega), and luciferase activity for each well as normalized to the internal renilla luciferase activity.

### Quantification of swimming, maximum velocity, and pharyngeal pumping

These methods are detailed elsewhere^64^,^65^. Briefly, for swimming movement, nematodes from a treatment group were randomly selected and transferred to a 6 cm petri dish containing 1 mL of M9 buffer. They were allowed to acclimate for ~10 s, then movements were scored for 30 seconds. Maximum velocity was determined as described previously^33^. N2 worms were exposed to designated concentrations of tomatidine (0, 10, 25, and 50 μM) beginning at L4 stage and maximum velocity of movement was determined on designated days. In brief, worms for imaging were moved to physical assay plate void of bacterial lawn. The recording system was a stereomicroscope (Unitron 2850), a charge-coupled device camera (Infinity 2, Lumenera), and imaging software. Four to eight nematodes were placed in one plate and recorded for 30 s, followed by data analysis using ImageJ and wrMTrck Batch (www.phage.dk/plugins).

For measurements of pharyngeal pumping (contractions) *C. elegans* were synchronized and raised to adults as mentioned in the longevity assay methodology. At designated ages pharynx contractions were manually counted for 30 s. Experiments were blinded and two or more researchers quantified pumping and swimming to decrease bias. Significance was calculated by student *t*-test.

### Age stage scoring of pharyngeal morphology

We imaged anesthetized worms, immobilized between an agarose pad and a number 1 coverslip, with an NA 1.4, 60x objective on an inverted Nikon Eclipse E800 microscope. The CCD output was saved as a stack and opened with ImageJ, for animals of a given cohort (drug treatment, age, etc.). A custom script was used to center a region of interest around the terminal bulb of each individual animal; the 300 × 300 pixel ROI was saved as a TIFF files, named systematically and later used to score the worms apparent age, as described^35^.

### C. elegans basal respiration

Basal oxygen consumption was measured per previous methods^66^ using a Seahorse XF96 instrument (Seahorse Bioscience Inc.). In brief, around 200 L4 nematodes/group were transferred to designated drug plates, followed by OCR detection on adult days 1 or 4. On the day of the experiment, all the *C. elegans* were collected using M9 buffer washing, followed by washing with M9 buffer twice. Nematodes were then re-suspended and transferred in 96-well standard Seahorse plates (#100777–004) (10~15 worms per well), and basal OCR was measured six times. After the experiments, nematodes numbers were counted, followed by data normalization to the number of nematodes in each individual well. We had ten replicates for each group, and repeated the experiment twice.

### Worm muscle mitochondrial network scoring

We used a *myo-3::gfp* reporter strain for mitochondrial networking morphology analysis in the body wall muscles. Worms were treated with the designated drugs from L4 stage for 4 days followed by imaging with a Zeiss confocal microscopy. We imaged over 15 worms/group each time (repeated two to three times). Mitochondrial network was evaluated in a double-blinded manner. A scoring scale of 1 to 5 was used, where a score of 5 denotes a perfectly organized mitochondrial network with healthy mitochondria running parallel with the myofilament lattice. For highly fragmented and disorganized mitochondrial network morphology, a score of 1 was given.

### RNA interference in *C. elegans*

RNA interference (RNAi) was performed as we did previously^67^. RNAi feeding constructs for L4440 (control), *dct-1, pink-1*, and *atfs-1* were derived from two RNAi feeding libraries (Dr. Sige Zou’s laboratory (previously form NIA) and the other one from Open Biosystems [the *E. coli* host strain is HT115 (DE3) (Fire Lab)]). RNAi was performed with worms maintained on the RNAi from egg laying stage throughout the experiment^67^.

### Metabolomics for *C. elegans* tissues

Whole worm tissue metabolomics were assessed with a method as described previously^67^. To evaluate changes of metabolome in N2 (veh) and N2 (tomatidine) worms, whole body tissues of adult Day 7 worms were collected and subjected to ALEX-CIS GCTOF mass spectrometry, UC Davis Genome Center Core Facilities. Four replicates were prepared for each group. Data were then normalized to internal control, followed by heat-map and TCR cycle analysis.

### Stress assays

Stress assays were conducted as described previously^5^. Briefly, *C. elegans* were treated with different doses of tomatidine, followed by exogenous stress exposure including either 25 μM rotenone for two days or exposure to 37 °C for 7 h. Nematodes were then evaluated for survival. Significance was calculated by student t-test.

### RNA isolation and Microarray

N2 *C. elegans* from L4 stage were exposed to either vehicle (1% DMSO) or 25 μM tomatidine (Sigma-Aldrich, USA). On adult day 7, nematodes where collected by washing off the plates with M9 buffer from four independent experiments. Samples were stored at −80 °C in lysis reagent (Qiagen Cat. no. 75142). Crude extracts were prepared by bead beating packed nematode pellets with the Mini-Beadbeater 8 (Biospec Products, Oklahoma), and total RNA isolated using RNeasy Mini Kit on the QIAcube per the manufacturer’s protocol (Qiagen, California). RNA concentration and quality was measured by Nanodrop (ThermoFisher, Waltham, MA USA) and the Agilent Bioanalyzer RNA 6000 Chip (Agilent, Santa Clara, CA). Two-hundred ng total RNA was labeled using the Agilent Low-Input QuickAmp Labeling Kit, and purified and quantified per the manufacturer’s instructions. A total of 825 ng Cy3-labeled cRNA was hybridized for 17 hours to Agilent *C. elegans* 4 × 44 K oligo microarrays. Following posthybridization rinses, arrays were scanned using an Agilent SureScan microarray Scanner at 3 micron resolution, and hybridization intensity data extracted from the scanned images using Agilent’s Feature Extraction Software. Raw microarray hybridization intensity data from four separate experiments were log-transformed. Three separate probe preparations were used individually or combined in equal amounts for the fourth hybridization. Raw microarray data were log transformed to yield z-scores. The z-ratio was calculated as the difference between the observed gene z-scores for the experimental and the control comparisons, and dividing by the standard deviation associated with the distribution of these differences. Z-ratio values of ±1.5 were used as cut-off values and calculated using a 5% false discovery rate (FDR) threshold. The complete set was tested for gene set enrichment using parametric analysis of gene set enrichment. For each pathway z-score, a p-value was computed using JMP 6.0 software to test for the statistical significance.

### Detection of mitophagy in cells and *C. elegans*

Mitophagy in nematodes was measured using a mitophagy reporter strain *N2;Ex(pmyo-3::dsred::lgg-1;pdct-1::dct-1::gfp)*. Nematodes were exposed to tomatidine at L4 stage, followed by imaging of muscle cells at adult D4. A total of 15~20 nematodes/group were imaged in three independent replicates. Colocalization coefficient between DSRED::DCT1 and LGG1::GFP were analyzed using the Zeiss LSM Image Examiner.

For mitophagy evaluation in cultured cells, we used different cell systems, including primary rat cortical neurons, human SH-SY5Y neural cells, and the mt-mKeima HeLa cells. A commercial mitophagy kit was used for mitophagy detection in both neurons and SH-SY5Y cells (Dojindo Laboratories). Imaging of mt-mKeima cells (gift from Dr. Richard Youle) was performed as reported by Nuo and colleagues^41^, using different settings for GFP (alkaline condition, excitation 458 nm and emission 615 nm) and RFP (acidic condition, excitation 561 nm and emission 615 nm).

### Statistics

For the worm lifespan analysis, the log rank test was used for statistical analysis. For two group comparison, two tailed student t-test was applied. GraphPad Prism 5 (GraphPad Software, Inc.) was used for statistical analyses. The *p* values < 0.05 were considered statistically significant.

## Additional Information

**How to cite this article**: Fang, E. F. *et al*. Tomatidine enhances lifespan and healthspan in *C. elegans* through mitophagy induction via the SKN-1/Nrf2 pathway. *Sci. Rep.* **7**, 46208; doi: 10.1038/srep46208 (2017).

**Publisher's note:** Springer Nature remains neutral with regard to jurisdictional claims in published maps and institutional affiliations.

## Supplementary Material

Supplementary Figure

Supplementary Table

Supplementary Table
