# Supplementary material for: Tomatidine enhances lifespan and healthspan in C. elegans through mitophagy induction via the SKN-1/Nrf2 pathway
Source: Sci Rep. 2017 Apr 11;7:46208. doi: 10.1038/srep46208 (PMC5387417; doi:10.1038/srep46208)
Supplement: Supplementary Figure [file srep46208-s1.pdf]

Supplementary Figures

Tomatidine enhances lifespan and healthspan in *C. elegans* through mitophagy induction via the SKN-1/Nrf2 pathway

Evandro F. Fang[1,6], Tyler B. Waltz[1,6], Henok Kassahun[1,2], Qiping Lu[1], Jesse S. Kerr[1], Marya Morevati[1,3], Elayne M. Fivenson[1], Bradley N. Wollman[1], Krisztina Marosi[4], Mark A. Wilson[4], Wendy B. Iser[4], D. Mark Eckley[5], Yongqing Zhang[5], Elin Lehrmann[5], Ilya G. Goldberg[5], Morten Scheibye-Knudsen[1,3], Mark P. Mattson[4], Hilde Nilsen[2], Vilhelm A. Bohr[1,3*], Kevin G. Becker[5,*]

[1]Laboratory of Molecular Gerontology, National Institute on Aging, National Institutes of Health, Baltimore, MD 21224
[2]Institute of Clinical Medicine, University of Oslo and Akershus University Hospital, 1478 Lørenskog, Norway
[3]Danish Center for Healthy Aging, University of Copenhagen, Blegdamsvej 3B, 2200 Copenhagen, Denmark
[4]Laboratory of Neurosciences, National Institute on Aging, National Institutes of Health, Baltimore, MD 21224
[5]Laboratory of Genetics and Genomics, National Institute on Aging, National Institutes of Health, Baltimore, MD 21224
[6] These authors contributed equally to this work.

[*]Correspondence should be addressed to VAB (BohrV@grc.nia.nih.gov) or KGB (beckerk@grc.nia.nih.gov), both are senior authors.

Figure S1. Effects of tomatidine on lifespan, maximum velocity and pharynx morphology.
(A) Effects of 50 μM tomatidine on the lifespan of N2 worms (*p* = 0.0495). *p*-values were calculated using the log-rank test, from pooled populations of animals (n = 86-98 worms/group).
(B) Table of the different lifespan replicates of 25μM tomatidine treated N2 *C. elegans* (n = 91-126). *p*-values were calculated using the log-rank test as done in Figure S1A.
(C) Effects of tomatidine on maximum velocity (n = 14-22 worms/group, Day 10 worms).
(D) Effects of different doses of tomatidine on the pharynx morphology in different age stages. A total of 120 worms/group were imaged. See Figure 1 for quantification.
(E) Representation of the “no damage”, “minor damage”, and “major damage” classifications used for actin filament analysis derived from phalloidin staining. White lines indicate gaps or disruptions in the actin filament structure. Demarcation between

minor and major damage is severity of these gaps, while the no damage category had virtually no disruption.
(F) Effects of tomatidine on the muscle morphology in day 4 and day 8 adult worms. The muscle cells were stained with phalloidin, then categorized as possessing “no damage”, “minor damage”, or “major damage”. The experiments were performed in two biological replicates, with 26~64 muscle cells imaged from 8~12 worms/repeat.

Figure S2. Tomatidine changes cell metabolism in worms, modifies mitochondrial parameters in primary neurons, and increases mitophagy in worms.
(A) Effects of tomatidine on the metabolism of metabolites and TCA cycle. Differences in metabolite levels between tomatidine-treated and control groups are denoted. A dash indicates no change. All the up-regulated or down-regulated data shown in the figure were with statistical significance compared with that of N2 (veh). Data were from four replicates.
(B-D) Effects of tomatidine (24 h) on mitochondrial content (B), mitochondrial membrane potential (C), and cellular ROS (D) in primary rat cortical neurons. MitoTracker Green (50 nm for 30 min) for mitochondrial content, TMRM (40 nM for 15 min) to detect mitochondrial membrane potential, and DCF-DA (20 µM for 30 min) to detect cellular ROS. Bar graphs are expressed as mean ± S.E.M. (n=24, and repeated two times).
(E) Quantification of the colocalization of LGG1/DCT-1 in vehicle and tomatidine-treated worm muscle cells (Correlated with Figure 3E).

Figure S3. No detectable effects of tomatidine on SIRT1 or PGC-1α promoter activities.
(A) Assessment of SIRT1 reporter activity in control and tomatidine-treated HEK-293 cells (mean ± S.E.M., n = 6, repeated twice)
(B) Assessment of PGC-1α promoter activity in control and tomatidien-treated HEK-293 cells (mean ± S.E.M., n = 6 and repeated twice)

Figure S4. Original blots.
(A) Full-length blots for Figure 3D.
(B) Full-length blots for Figure 5B.

Fig. S1

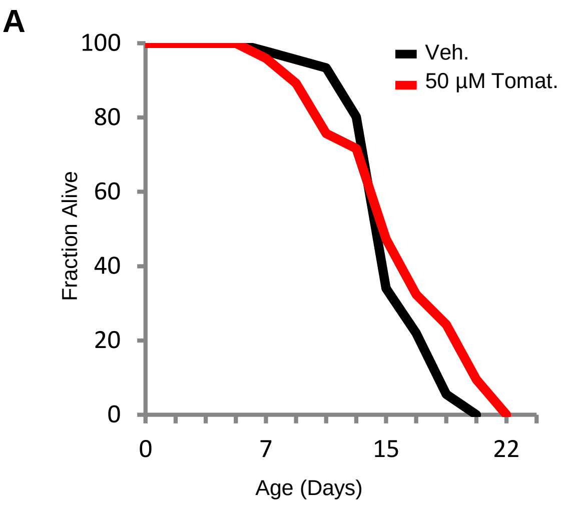

B

Complete Lifespan Data	Vehicle Mean Lifespan (Days ± SEM)	25µM Tomatidine Mean Lifespan (Days ± SEM)	*p*-value	Sample Size (Vehicle, Treatment)
Trial 1	15.2 ± 0.3	16.3 ± 0.3	<0.001	91, 91
Trial 2	16.1 ± 0.3	17.1 ± 0.3	0.013	112, 115
Trial 3	16.5 ± 0.3	17.8 ± 0.3	<0.001	112, 126

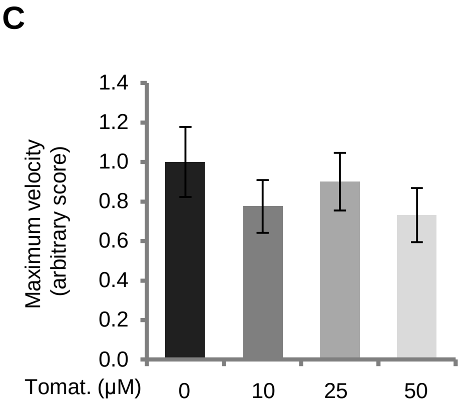

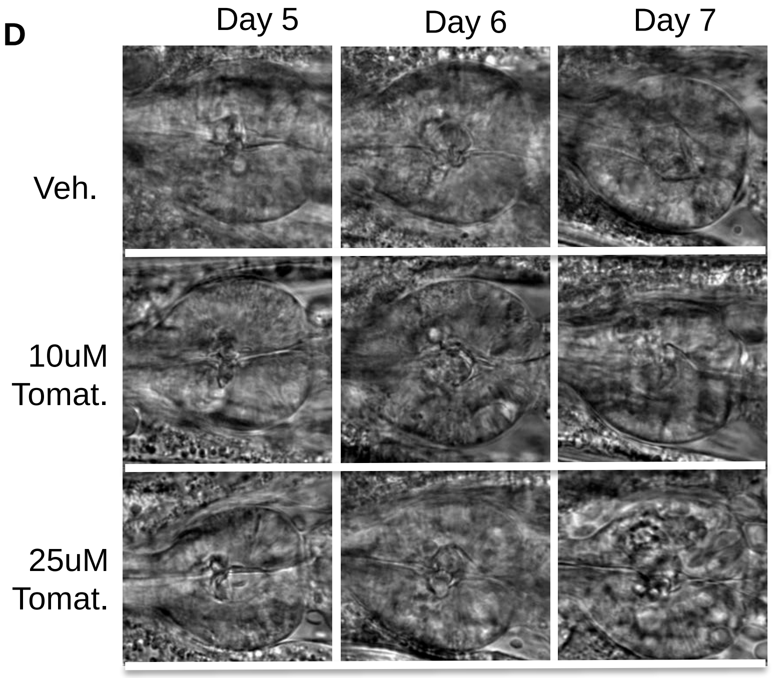

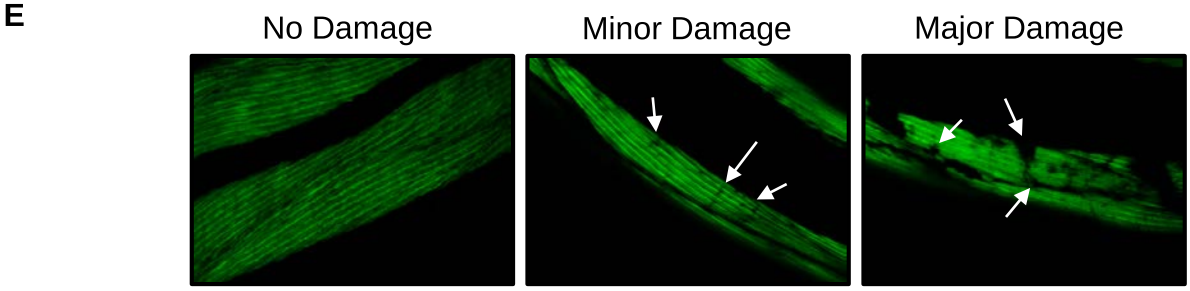

F

	N2			N2 w/ 25µM tomatidine		
**Day 4**	No Dmg.	Minor Dmg.	Major Dmg.	No Dmg.	Minor Dmg.	Major Dmg.
Trial 1	52%	46%	2%	44%	56%	0%
Trial 2	66%	33%	0%	79%	19%	2%

	N2			N2 w/ 25µM tomatidine		
**Day 8**	No Dmg.	Minor Dmg.	Major Dmg.	No Dmg.	Minor Dmg.	Major Dmg.
Trial 1	4%	42%	54%	1%	75%	24%
Trial 2	7%	78%	15%	0%	93%	7%

Fig. S2

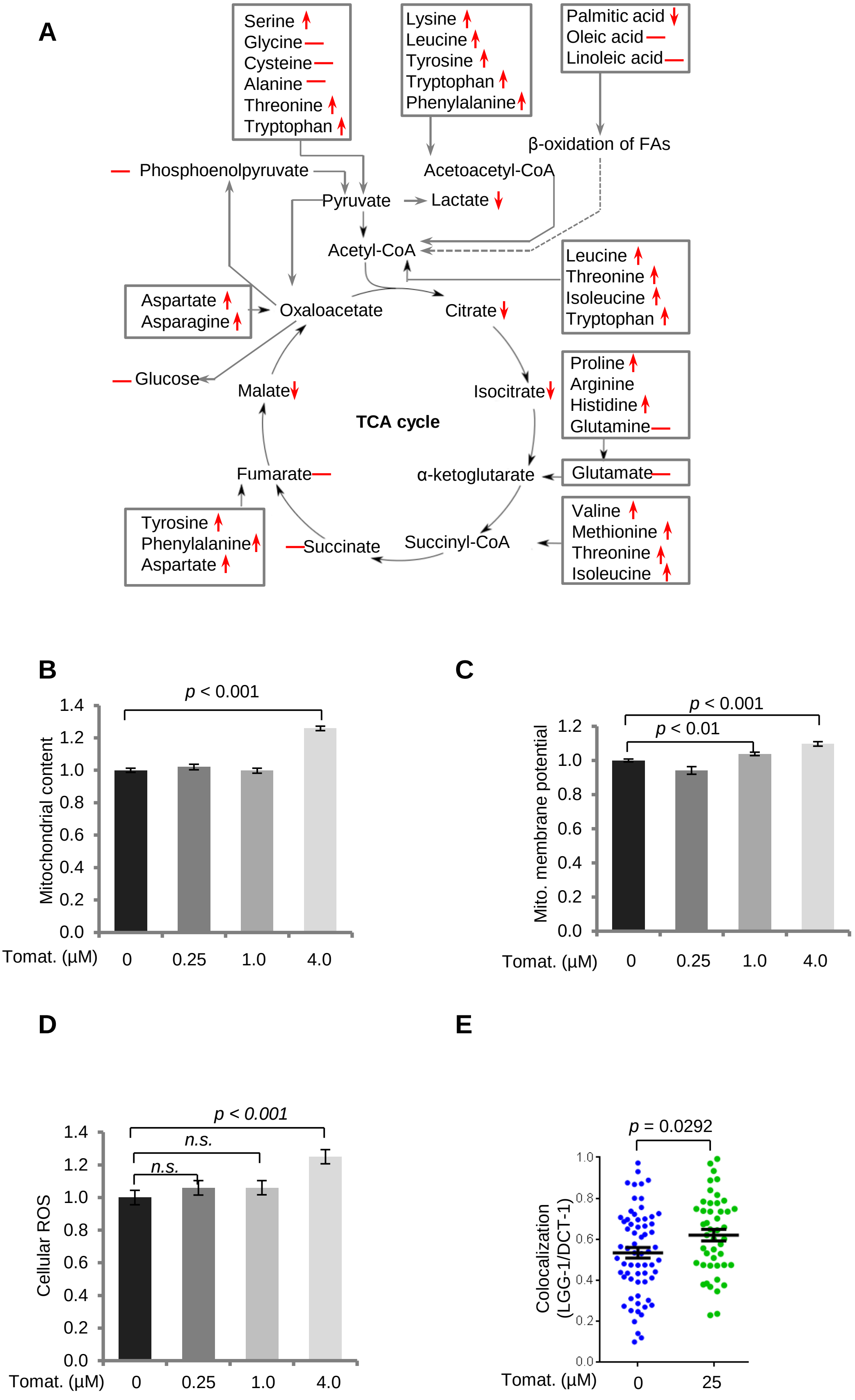
A
Serine
Glycine
Cysteine
Alanine
Threonine
Tryptophan
Lysine
Leucine
Tyrosine
Tryptophan
Phenylalanine
Palmitic acid
Oleic acid
Linoleic acid
β-oxidation of FAs
Phosphoenolpyruvate
Acetoacetyl-CoA
Pyruvate
Lactate
Acetyl-CoA
Leucine
Threonine
Isoleucine
Tryptophan
Aspartate
Asparagine
Oxaloacetate
Citrate
Glucose
Malate
Isocitrate
Proline
Arginine
Histidine
Glutamine
TCA cycle
Fumarate
α-ketoglutarate
Glutamate
Valine
Methionine
Threonine
Isoleucine
Tyrosine
Phenylalanine
Aspartate
Succinate
Succinyl-CoA
B
p < 0.001
Mitochondrial content
1.4
1.2
1.0
0.8
0.6
0.4
0.2
0.0
Tomat. (μM)
0
0.25
1.0
4.0
C
p < 0.001
p < 0.01
Mito. membrane potential
1.2
1.0
0.8
0.6
0.4
0.2
0.0
Tomat. (μM)
0
0.25
1.0
4.0
D
p < 0.001
n.s.
n.s.
Cellular ROS
1.4
1.2
1.0
0.8
0.6
0.4
0.2
0.0
Tomat. (μM)
0
0.25
1.0
4.0
E
p = 0.0292
Colocalization
(LGG-1/DCT-1)
1.0
0.8
0.6
0.4
0.2
0.0
Tomat. (μM)
0
25

Fig. S3

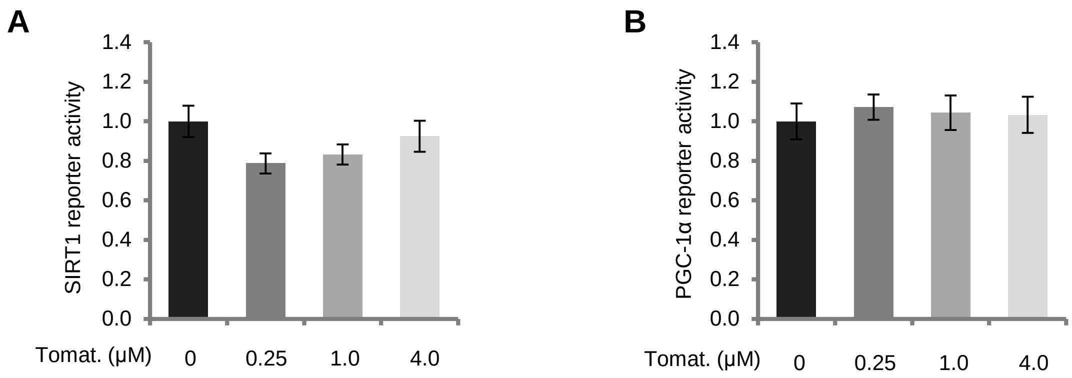
A
SIRT1 reporter activity
1.4
1.2
1.0
0.8
0.6
0.4
0.2
0.0
Tomat. (μM)
0
0.25
1.0
4.0
B
PGC-1α reporter activity
1.4
1.2
1.0
0.8
0.6
0.4
0.2
0.0
Tomat. (μM)
0
0.25
1.0
4.0

Fig. S4

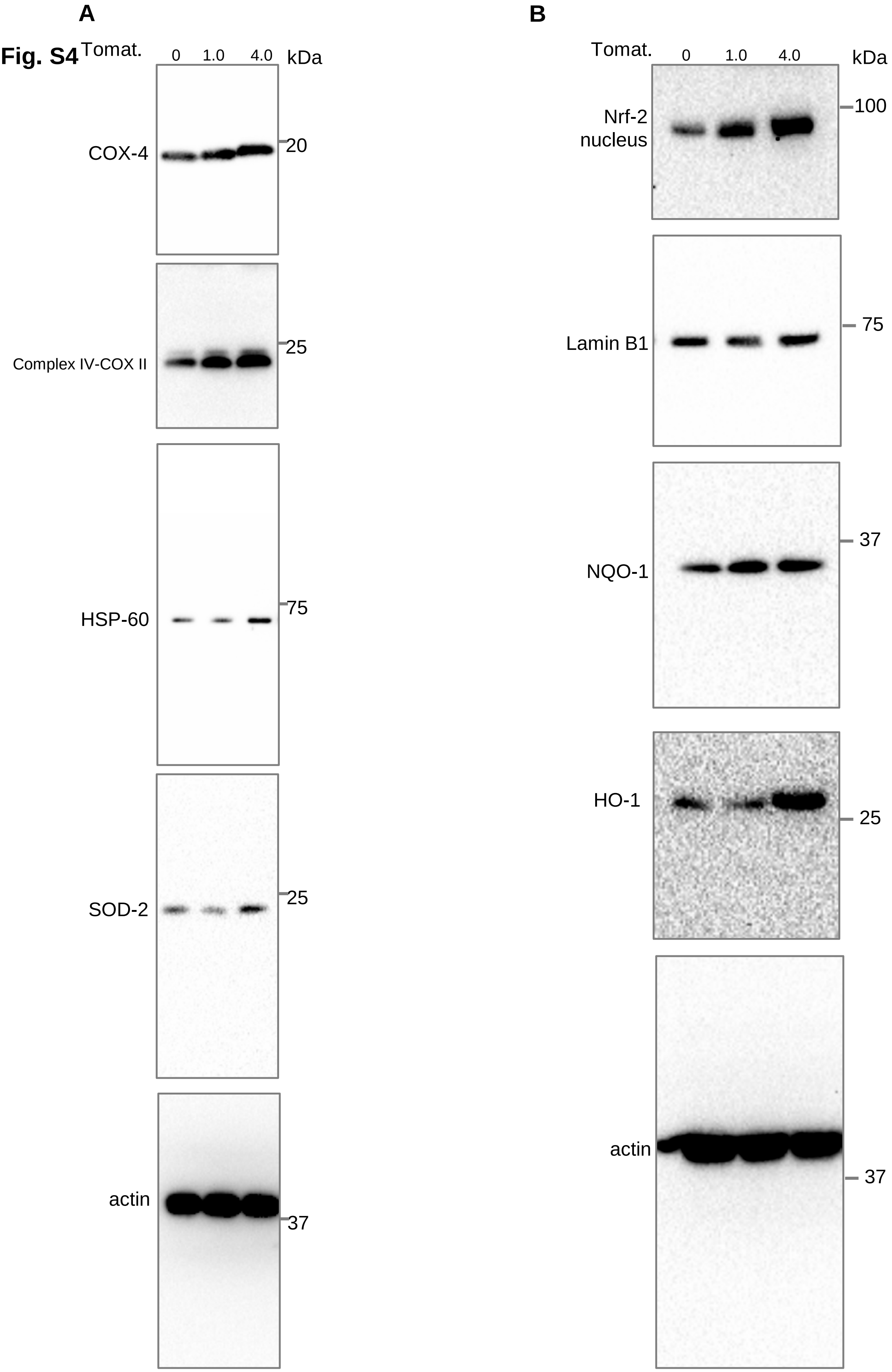
A
Tomat.
0
1.0
4.0
kDa
COX-4
20
Complex IV-COX II
25
HSP-60
75
SOD-2
25
actin
37
B
Tomat.
0
1.0
4.0
kDa
Nrf-2
nucleus
100
Lamin B1
75
NQO-1
37
HO-1
25
actin
37
